# Supplementary figures and images for: Legume integration in smallholder farming systems for food security and resilience to climate change
Source: PLoS One. 2025 Aug 13;20(8):e0327727. doi: 10.1371/journal.pone.0327727 (PMC12349721; doi:10.1371/journal.pone.0327727)

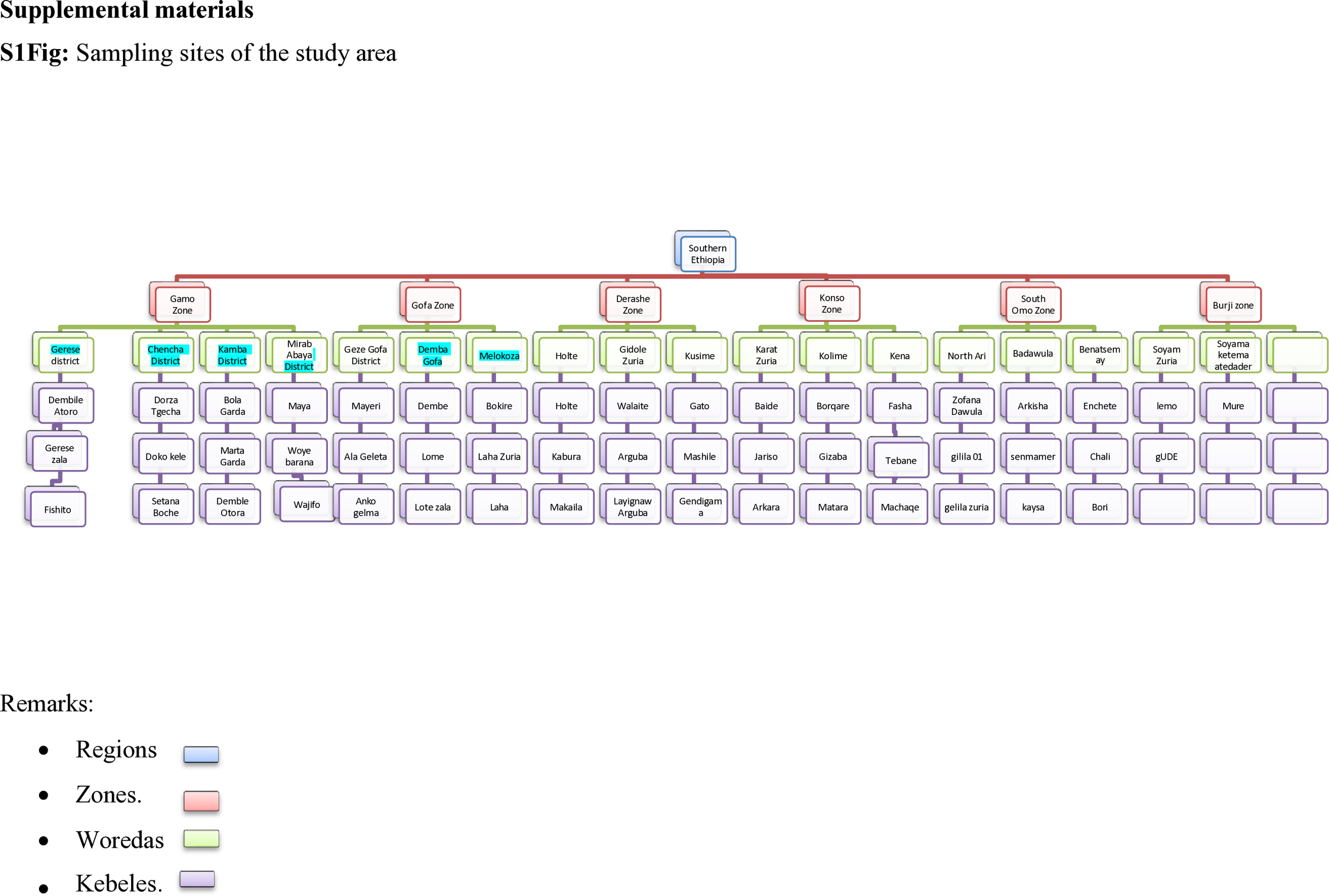

Supplement: S1 Fig — (TIF) [file pone.0327727.s001.tif]

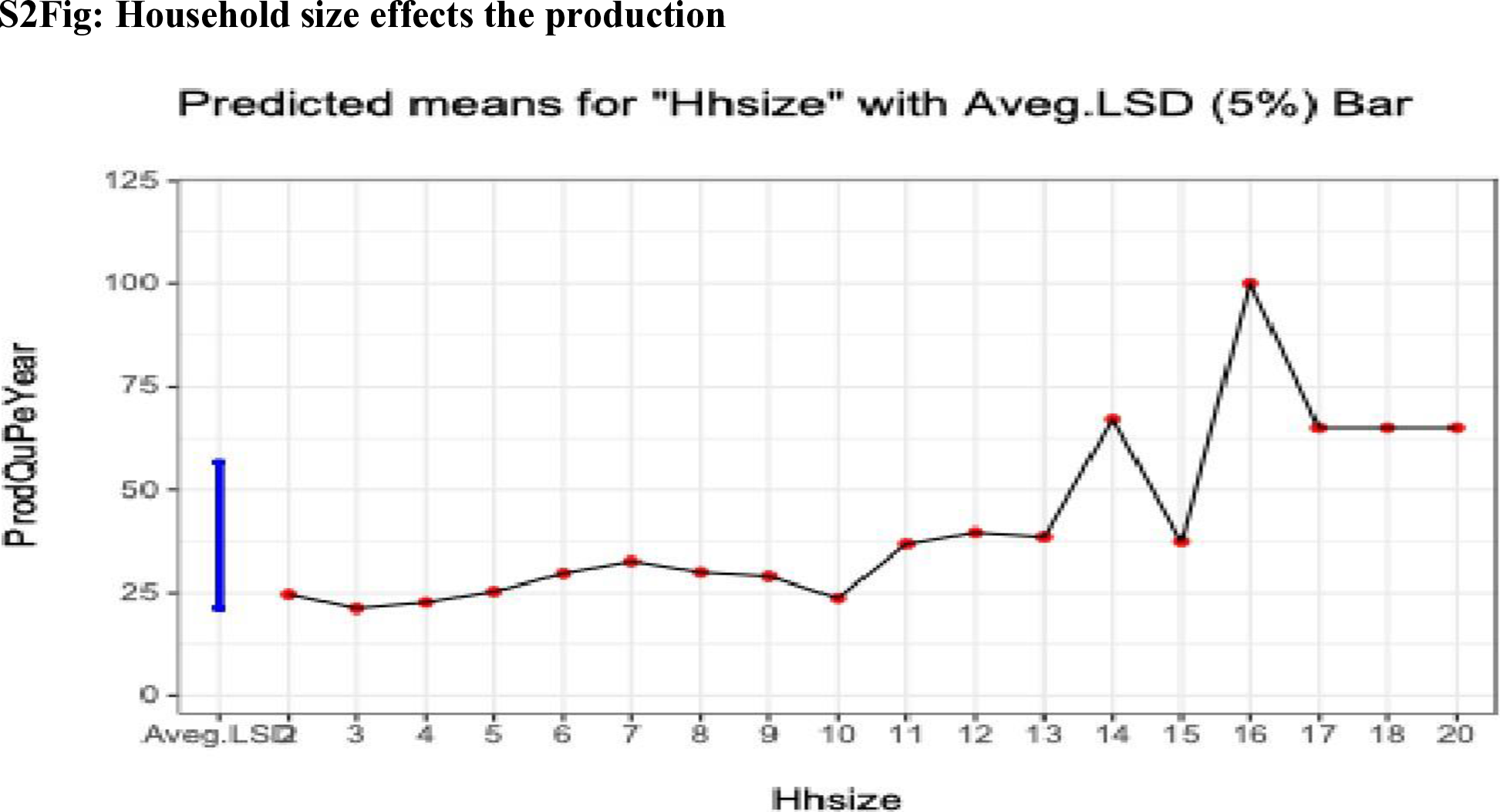

Supplement: S2 Fig — (TIF) [file pone.0327727.s002.tif]

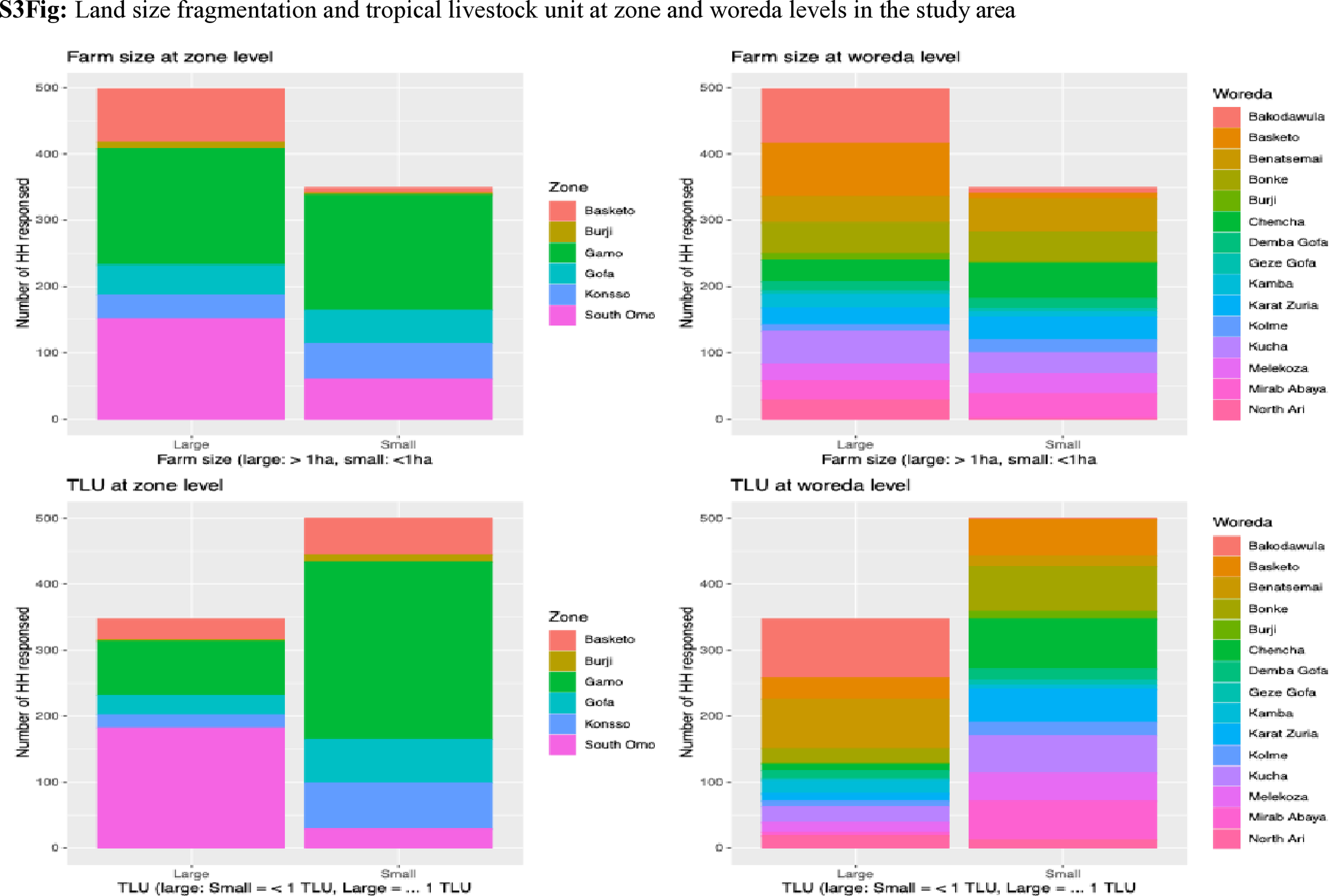

Supplement: S3 Fig — (TIF) [file pone.0327727.s003.tif]

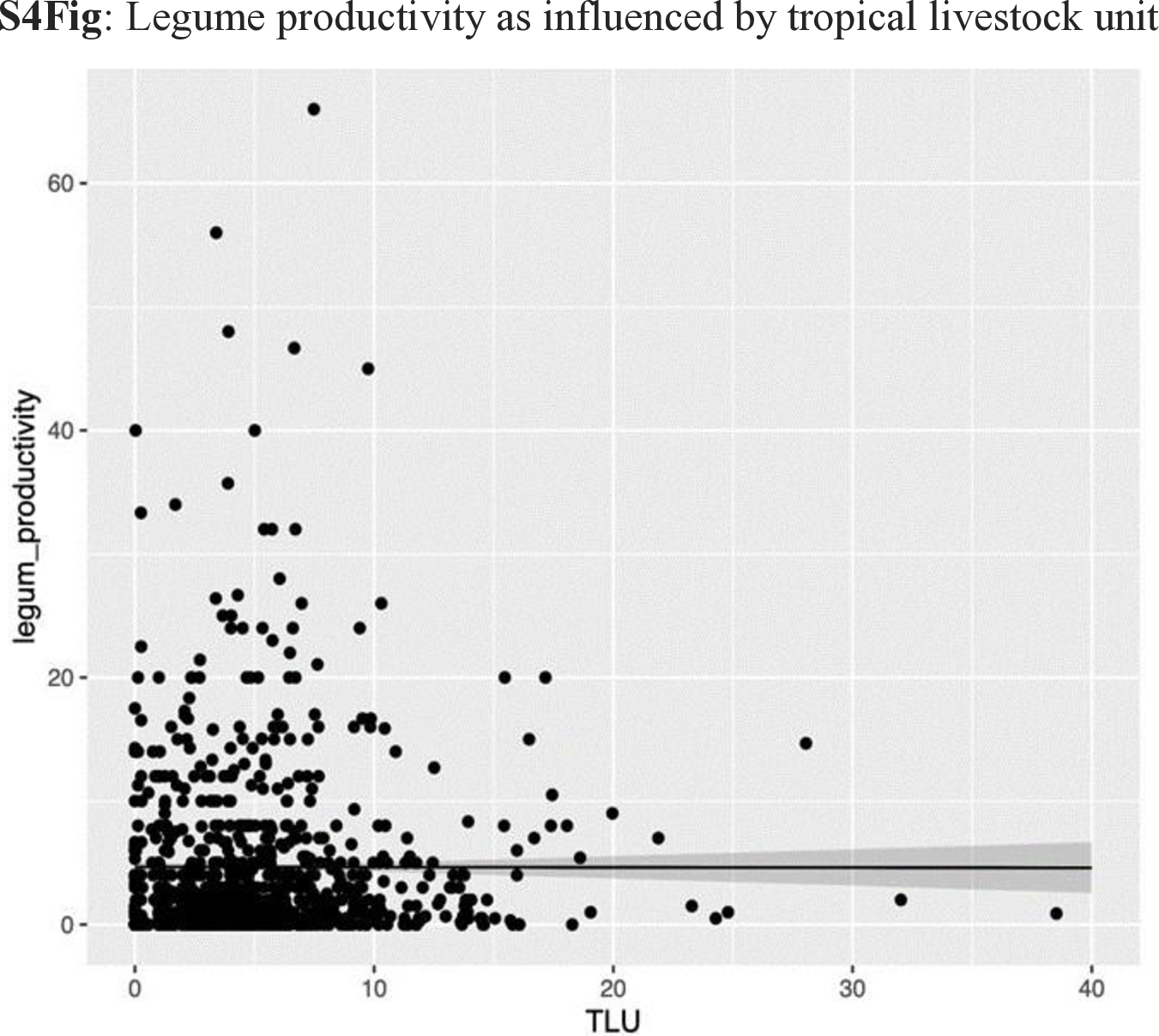

Supplement: S4 Fig — (TIF) [file pone.0327727.s004.tif]

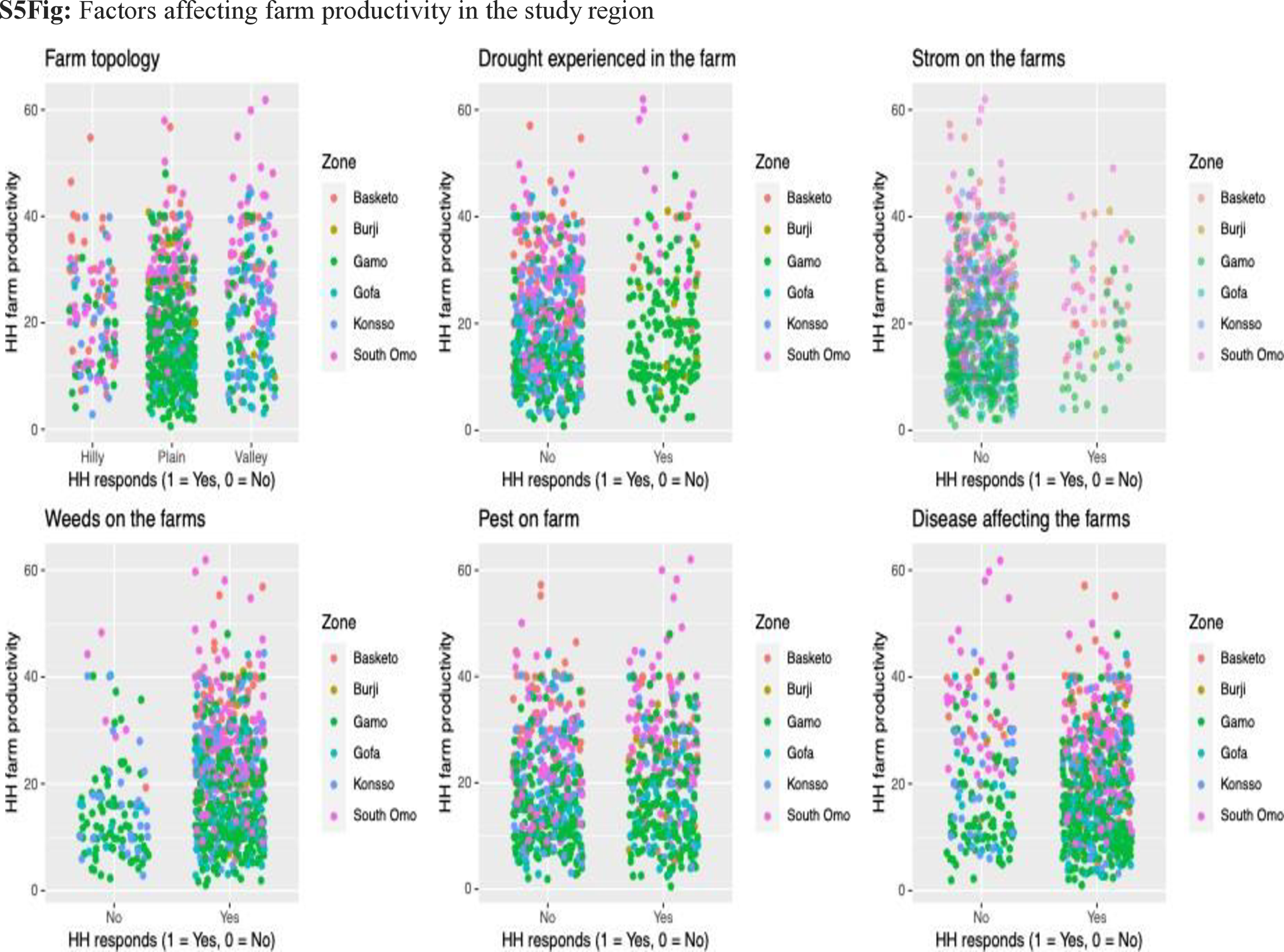

Supplement: S5 Fig — (TIF) [file pone.0327727.s005.tif]

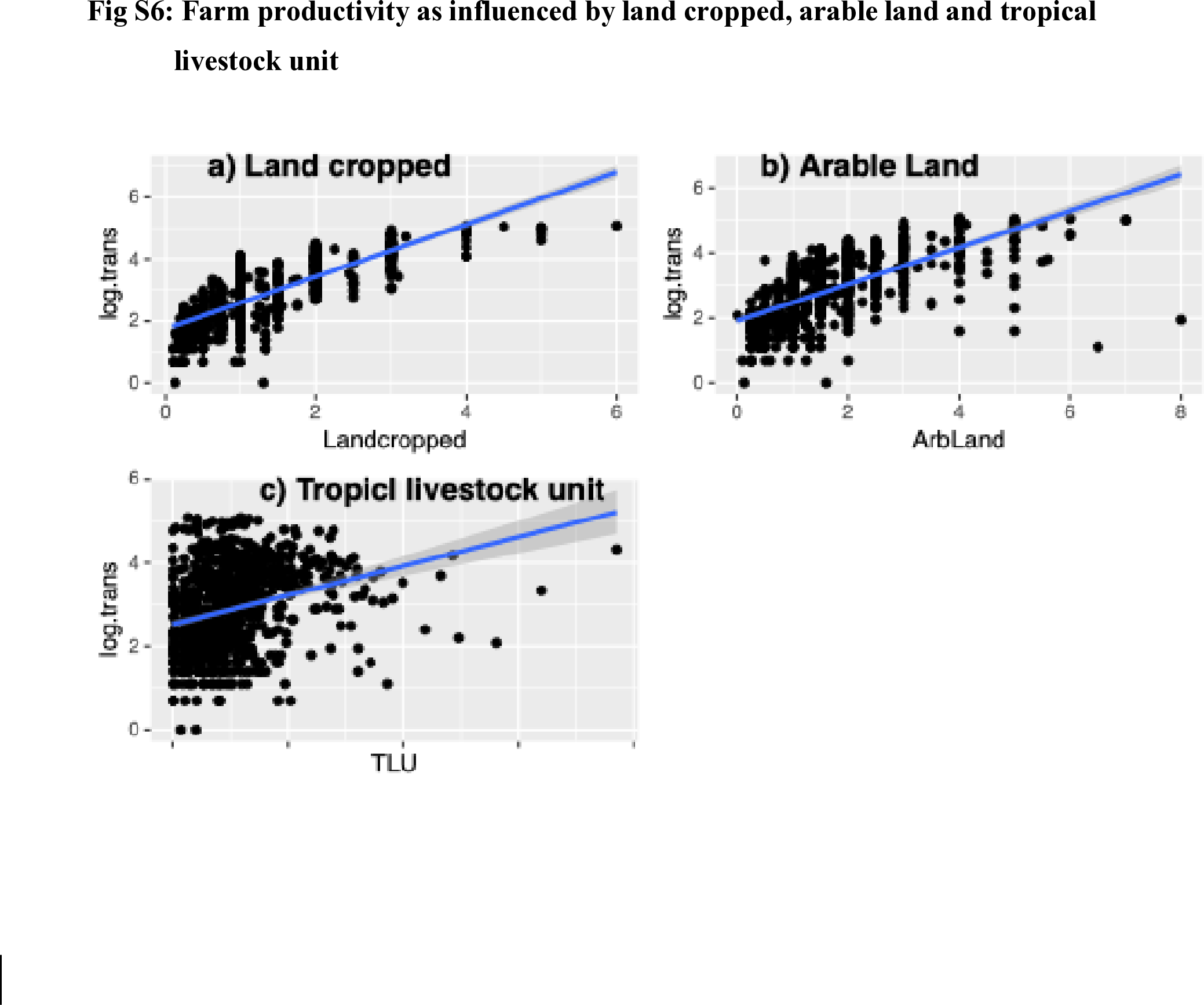

Supplement: S6 Fig — (TIF) [file pone.0327727.s006.tif]

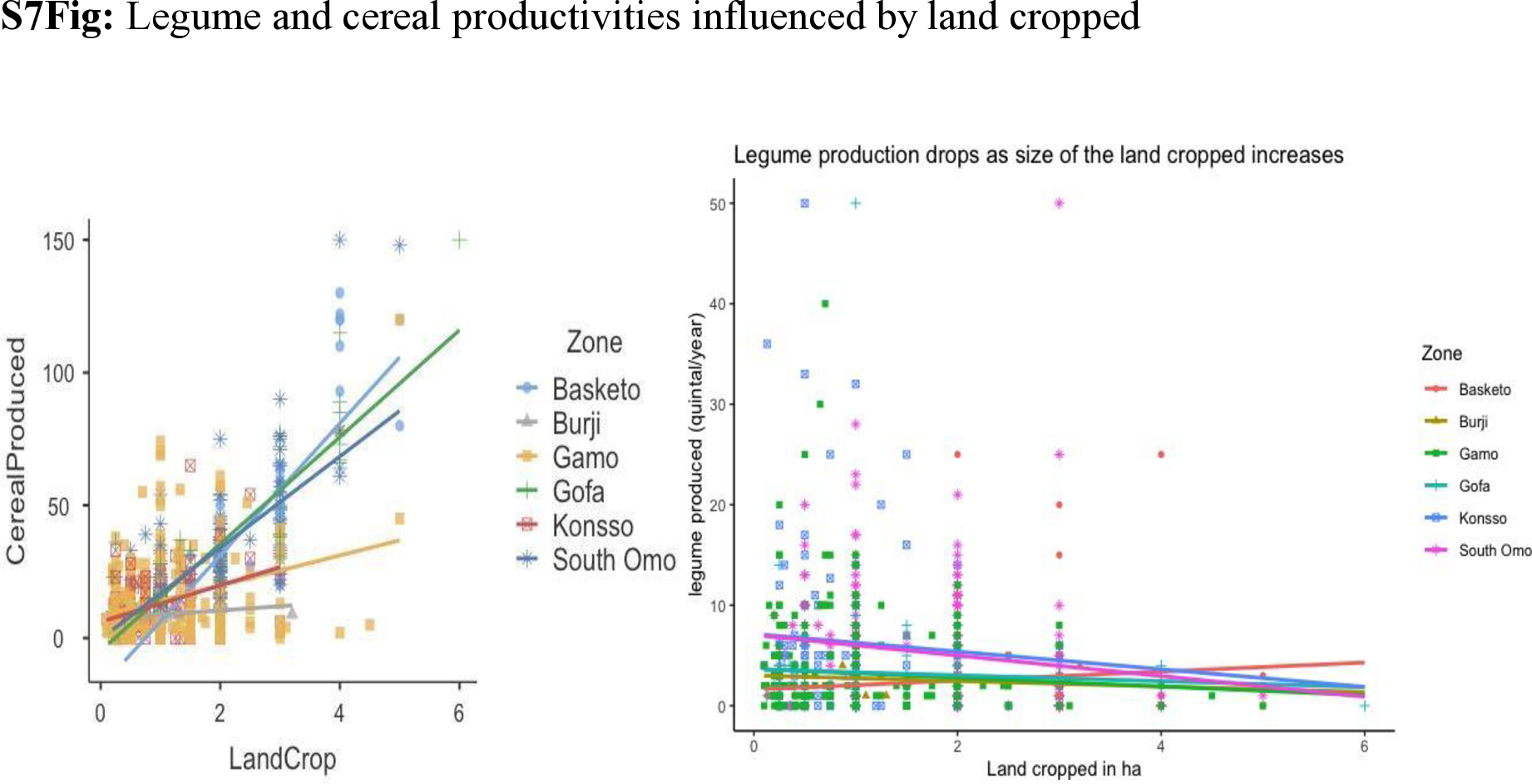

Supplement: S7 Fig — (TIF) [file pone.0327727.s007.tif]

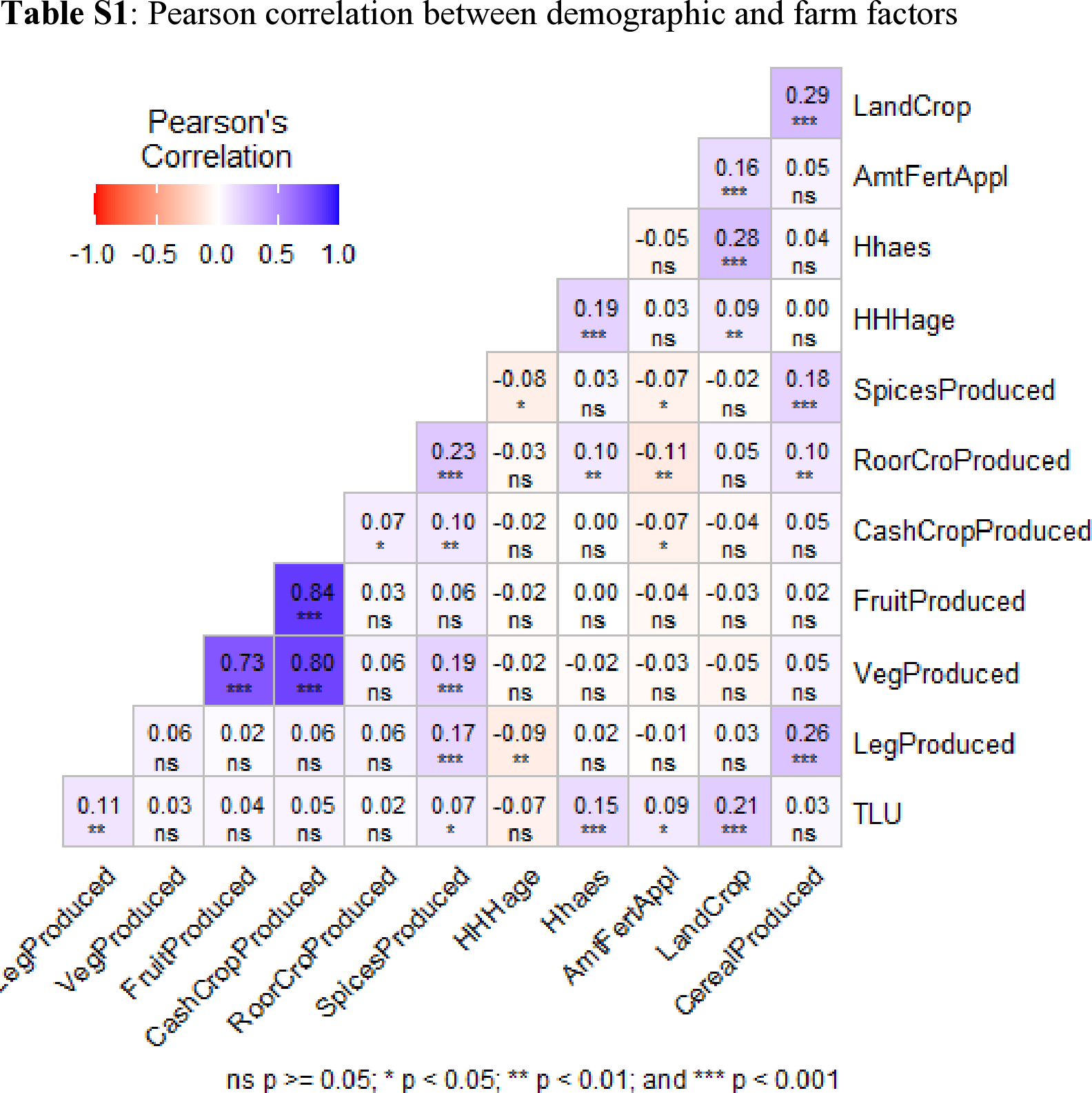

Supplement: Table S1 — (TIF) [file pone.0327727.s008.tif]
