# Supplementary material for: Legume integration in smallholder farming systems for food security and resilience to climate change
Source: PLoS One. 2025 Aug 13;20(8):e0327727. doi: 10.1371/journal.pone.0327727 (PMC12349721; doi:10.1371/journal.pone.0327727)
Supplement: Attachment — Submitted filename: Reviewer Response .docx [file pone.0327727.s009.docx]

| Reviewer Questions | Response |
| --- | --- |
| Please provide additional details regarding participant consent. In the ethics statement in the Methods and online submission information, please ensure that you have specified (1) whether consent was informed and (2) what type you obtained (for instance, written or verbal, and if verbal, how it was documented and witnessed). If your study included minors, state whether you obtained consent from parents or guardians. If the need for consent was waived by the ethics committee, please include this information. | **Ethical considerations**   - Before starting the study, ethical approval was sought and granted by the Institutional Review Board of Arba Minch University with the reference number CNS/FO29/2022. The response obtained for this specific section was deemed as 'Not applicable'. Formal authorization was obtained from the respective agriculture and rural development offices for commencing activities in each zone and district. The research protocol was thoroughly elucidated to the participants with agricultural knowledge to solicit their written approval prior to engaging in the study, and written consent was duly acquired from these individuals. Additionally, detailed information regarding the household heads with agricultural expertise was collected. Throughout the process of the study privacy, anonymity, and confidentiality were ensured. Household heads were informed that they have the right to refuse from giving consent and withdraw from the study at any time. Those households with food insecurity were reported to zonal and district agricultural and rural development office. During the data collection process, local language was used to communicate with each household heads. - The consent format was carefully prepared in a written format in the English language, subsequently translated into Amharic (the working language of the region), and thereafter printed and dispatched to data collectors. In instances where the target farmers are literate (capable of comprehending the consent document), they affixed their signatures on two copies (one copy retained within each kebele, while the other is preserved at AMU). For individuals who are illiterate and unable to communicate in Amharic, the consent document was translated into their indigenous language, allowing them to provide their signature thereon. - With respect to the inclusion of minors, our research does not involve individuals under the age of 18; all participants are smallholder farmers whose ages range from 21 to 83. Each participant is the head of their household and has provided their own consent. |
| Please remove any funding-related text from the manuscript. | Funding related text was only available in the acknowledgement section; it was removed**.** |
