# Supplementary material for: Legume integration in smallholder farming systems for food security and resilience to climate change
Source: PLoS One. 2025 Aug 13;20(8):e0327727. doi: 10.1371/journal.pone.0327727 (PMC12349721; doi:10.1371/journal.pone.0327727)
Supplement: Attachment — Submitted filename: Response to reviewers -.docx [file pone.0327727.s011.docx]

Response to Reviewers letter

| Points raised by academic editors/reviewers | Author response |
| --- | --- |
| 1. Reviewer #1: Still i am not satisfied with the food security measurement applied and the linkage with resilience to climate change. I prefer if it would have been consider the temperature and rainfall in relation to food security. without considering the temperature and rainfall trends considering other parameter to see the linkage between food security and resilience to climate change is incomplete | Dear Reviewer thank you for your concern, as noted. We integrated the link between rising temperatures and the consequences of rainfall variability on food security, as mentioned in lines 604-614. |
| 2. Reviewer #3: The abstract is informative and captures key findings effectively; however, there are minor grammatical inconsistencies, awkward phrasing, and issues with tense consistency that need revision for clarity and professionalism | Thank you for your input. We strive to remove grammatical problems online 16-27, 30, 32, 34 , 36, and completely improve the abstract part. |
| 3. It is recommended to clarify abbreviations on first use, refine vague expressions (such as "higher levels"), and maintain consistent past tense throughout. Strengthening the transitions between findings would also improve the abstract’s overall flow and readability. | Thank you for your input. It was rephrased on lines 18-20 |
| 4. • Adjust the phrasing about agricultural practices to maintain past tense ("some were found to negatively affect productivity"). | We appreciate your effort, and we rephrased it from lines 459-488. |
| 5. Some have been found to affect it negatively" → "some were found to affect it negatively" (tense consistency). | It was rephrased from lines 25 |
| 6. "Farmers have disclosed" → "farmers disclosed" (simpler, more formal past tense preferred). | Re phrased on line 33 |
| 7. Revise the sentence on gender and productivity to specify "higher productivity levels" and clarify resource utilization. | It was rephrased from lines 15 to 17, 247 |
| 8. Conclusions  The content provides valuable insights into household demographics, farm variability, and legume production challenges. However, I suggest merging the two paragraphs into a single coherent paragraph to improve the flow and readability. Additionally, please consider revising and splitting the following long sentence to enhance clarity:  • The sentence starting with "Even if, the region was rich in growing diverse crops." (lines 631–632) is too lengthy and could be broken into two sentences | We truly value your recommendation, which was rephrased in one paragraph. |
| 9. Results  Given that higher educational attainment among household heads did not lead to improved farm productivity, what factors might be limiting the effective translation of education into practical agricultural outcomes in this region? | It was justified from lines 219 to 221 |
| 10. The manuscript mentions that farmers who applied fertilizers did not necessarily achieve higher yields compared to those who did not. However, this statement lacks clarity and supporting data. Please revise this section to clearly explain whether the lower yields were statistically significant and specify whether factors such as crop type, fertilizer type, or application rates were controlled for in the comparison | The concept is clarified further, as restated in lines 314 to 323  The supporting data was reanalyzed on table 3, |
| 11. In the section discussing land fragmentation, you report both farmland expansion and shrinking farm sizes due to population growth, which appears contradictory. Please clarify this point: are farmers expanding into new lands while their individual farm holdings are simultaneously becoming smaller due to subdivision among heirs? A clearer explanation is needed to avoid confusion | The justification was added from line 468 to 470  Also we restated in discussion part from line 509-513 |
| 12. Methodology  The methodology is clearly described; however, could you clarify how data quality was ensured when combining digital and paper-based data collection methods? Specifically, was there any verification or cross-checking steps taken to minimize inconsistencies between the two approaches? | We add the justification from line 179 to 182 |
| 13. Some references are older (e.g., 2014–2017). Consider including more recent studies (2020–2024) to strengthen the background. | We carefully reviewed your suggestion regarding references, and it was substituted and updated on the reference number 3, 7, 8, 10, 12, 13, 14, 21, 26, 27, 28, 33, 34, 36, 39, are updated. |
