# Supplementary material for: Legume integration in smallholder farming systems for food security and resilience to climate change
Source: PLoS One. 2025 Aug 13;20(8):e0327727. doi: 10.1371/journal.pone.0327727 (PMC12349721; doi:10.1371/journal.pone.0327727)
Supplement: Attachment — Submitted filename: Reviewer comment.docx [file pone.0327727.s010.docx]

The abstract is informative and captures key findings effectively; however, there are minor grammatical inconsistencies, awkward phrasing, and issues with tense consistency that need revision for clarity and professionalism.

It is recommended to clarify abbreviations on first use, refine vague expressions (such as "higher levels"), and maintain consistent past tense throughout. Strengthening the transitions between findings would also improve the abstract’s overall flow and readability.

Please make the recommended changes to the following lines to improve clarity, grammatical accuracy, and consistency:

- Revise the sentence on gender and productivity to specify "higher productivity levels" and clarify resource utilization.
- Adjust the phrasing about agricultural practices to maintain past tense ("some were found to negatively affect productivity").
- "some have been found to affect it negatively" → "some were found to affect it negatively" (tense consistency).
- "farmers have disclosed" → "farmers disclosed" (simpler, more formal past tense preferred).

**Conclusion**

The content provides valuable insights into household demographics, farm variability, and legume production challenges. However, I suggest merging the two paragraphs into a single coherent paragraph to improve the flow and readability. Additionally, please consider revising and splitting the following long sentence to enhance clarity:

- The sentence starting with *"Even if, the region was rich in growing diverse crops..."* (lines 631–632) is too lengthy and could be broken into two sentences.

Overall, the findings are relevant, but these improvements will make the presentation much clearer.

**Results**

Given that higher educational attainment among household heads did not lead to improved farm productivity, what factors might be limiting the effective translation of education into practical agricultural outcomes in this region?

The manuscript mentions that farmers who applied fertilizers did not necessarily achieve higher yields compared to those who did not. However, this statement lacks clarity and supporting data. Please revise this section to clearly explain whether the lower yields were statistically significant and specify whether factors such as crop type, fertilizer type, or application rates were controlled for in the comparison.

In the section discussing land fragmentation, you report both farmland expansion and shrinking farm sizes due to population growth, which appears contradictory. Please clarify this point: are farmers expanding into new lands while their individual farm holdings are simultaneously becoming smaller due to subdivision among heirs? A clearer explanation is needed to avoid confusion.

**Methodology**

The methodology is clearly described; however, could you clarify how data quality was ensured when combining digital and paper-based data collection methods? Specifically, were there any verification or cross-checking steps taken to minimize inconsistencies between the two approaches?

**Reference**

Some references are older (e.g., 2014–2017). Consider including more recent studies (2020–2024) to strengthen the background.
